# Case Report: Repeated courses of low-frequency rTMS for refractory tinnitus: an N-of-1 study

**DOI:** 10.3389/fpsyt.2026.1870689

**Published:** 2026-07-31

**Authors:** Ardıl Bayram Şahin, Melike Kocahasan, Rışvan Deniz, Ahmet Ataş, Cem Uzun, Hale Yapici Eser

**Affiliations:** 1 Research Center for Translational Medicine (KUTTAM), Koç University, Istanbul, Türkiye; 2 Graduate School of Health Sciences, Koç University, Istanbul, Türkiye; 3 Department of Psychiatry, Biruni University Faculty of Medicine, Psychiatry, Istanbul, Türkiye; 4 Koç University School of Medicine, Istanbul, Türkiye; 5 Department of Audiology, Koç University Hospital, Istanbul, Türkiye; 6 Department of Otorhinolaryngology, Koç University School of Medicine, Istanbul, Türkiye; 7 Department of Psychiatry, Koç University School of Medicine, Istanbul, Türkiye

**Keywords:** chronic tinnitus, longitudinal assessment, neuromodulation, psychiatric burden, repetitive transcranial magnetic stimulation, single-case study

## Abstract

**Background:**

Chronic tinnitus is a disabling condition with limited long-term treatments, especially in refractory cases. Although repetitive transcranial magnetic stimulation (rTMS) shows short-term benefits, its sustained effects are unclear. This study evaluated long-term outcomes of repeated courses of rTMS in treatment-resistant chronic tinnitus.

**Methods:**

This retrospective single-subject case study followed a 30-year-old female with severe, treatment-resistant chronic tinnitus. Three identical courses of repetitive rTMS were administered over one year, each consisting of 10 sessions of 1 Hz stimulation applied to the left auditory cortex at 110% of the resting motor threshold. The patient remained medication-free throughout the study. Outcomes included tinnitus severity assessed by the Tinnitus Handicap Inventory (THI), Tinnitus Severity Index (TSI), and a tinnitus visual analog scale (VAS); psychiatric symptoms assessed by the Patient Health Questionnaire-9 (PHQ-9), Generalized Anxiety Disorder-7 (GAD-7), Chronic Stress Scale (CSS), and Symptom Checklist-90-Revised (SCL-90-R); and audiometric evaluations. Effect sizes were quantified using standardized mean change (SMC) indices, calculated as the change in score relative to the instrument’s normative standard deviation.

**Results:**

Across three rTMS courses, tinnitus severity progressively decreased: THI dropped from 98 to 50, TSI from 59 to 41, and VAS from 10 to 5. Although short-term responses varied, overall effects were large (SMC −2.00 to −2.34). Psychological symptoms also improved markedly, with the greatest changes after the first course followed by stabilization. Marked reductions were observed in depression (PHQ-9), anxiety (GAD-7), and stress measures. Global psychological distress (SCL-90-R) declined sustainably, including improvements in overall severity and symptom domains. Peripheral hearing thresholds remained stable throughout follow-up, indicating no adverse auditory effects.

**Conclusion:**

This case study suggests repeated low-frequency rTMS may be associated with reductions in tinnitus severity and psychiatric symptoms without medication. Observed changes varied with affective and stress states, indicating improvement may reflect reduced attentional bias rather than elimination of tinnitus. Findings warrant larger controlled studies of repeated courses of rTMS in chronic tinnitus.

## Background

Tinnitus is characterized by an abnormal auditory perception in the head or ears in the absence of any external acoustic stimulus. This sound is commonly described as humming, ringing, clicking, and other sounds. It is a common auditory symptom that causes significant psychological distress in patients and is frequently associated with comorbid symptoms such as hearing loss, dizziness, concentration difficulties, cognitive, emotional, and sleeping problems (1). A 2022 meta-analysis reported a global tinnitus prevalence of 14.4%, rising from 9.7% in adults aged 18–44 to 23.6% in those 65+, with no effect of sex. The prevalence of severe tinnitus was reported as 2.3%, chronic tinnitus as 9.8%, and diagnosed tinnitus as 3.4%, indicating substantial underdiagnosis. Many hypotheses have been proposed regarding the etiology of tinnitus, yet it has not been fully explained. Increased excitability of pyramidal and VIP neurons and reduced GABAergic inhibition in the primary auditory cortex were reported in rats with tinnitus-like behaviors (2). These excitatory-inhibitory imbalances correlated with tinnitus severity, consistent with the view that tinnitus is a maladaptive central plasticity process rather than purely peripheral damage. Currently, despite numerous approaches, no standard tinnitus treatment has been developed.

Neuromodulation techniques, including electrical and electromagnetic stimulation, are thought to attenuate pathological central hyperactivity by modulating cortical excitability, inhibitory-excitatory balance, and large-scale functional network dynamics (3). Repetitive transcranial magnetic stimulation (rTMS) is being investigated as a treatment for chronic tinnitus. A meta-analysis showed statistically significant reductions in THI scores following low-frequency (1 Hz) rTMS targeting the auditory cortex at 1-week, 1-month, and 6-month follow-up compared to sham stimulation, with evidence of short- to medium-term clinical benefit (1). However, updated evidence highlights considerable heterogeneity and uncertainty regarding the durability of these effects at longer follow-up intervals (4). A recent meta-analysis of randomized controlled trials demonstrated that low-frequency rTMS is associated with significant reductions in Tinnitus Handicap Inventory (THI) and visual analogue scale (VAS) scores immediately after treatment and at 1-month follow-up. However, the durability of these effects beyond 6 months remains uncertain, with no statistically significant long-term benefit observed (5). Retrospective data indicate response rates exceeding 60% (6). Placebo-controlled trials highlight superior outcomes with left temporoparietal stimulation. Improvements in tinnitus severity correlate with reduced anxiety (7).

In the current literature, most rTMS interventions for tinnitus employ relatively brief protocols (5–10 sessions), and although some studies have reported follow-up outcomes extending to 6–12 months, relatively little is known about the longitudinal effects of multi-courses of rTMS within the same individual. In addition, most studies focus primarily on tinnitus severity outcomes, whereas comprehensive assessments incorporating psychiatric symptoms, stress-related measures, and audiometric evaluations remain uncommon. Given the heterogeneity of tinnitus presentation and treatment response, a single-subject (N-of-1) approach allows detailed characterization of symptom trajectories and potential contextual influences over time. To address this gap, we report a retrospective N-of-1 case in which three identical 10-session rTMS courses were administered over one year. Rather than the follow-up duration per se, the distinctive features of this case are the repeated-course design and the integration of a broad multidimensional battery, allowing within-subject evaluation of psychiatric dimensions alongside tinnitus severity.

This retrospective single-subject framework was used to examine whether repeated courses of identical low-frequency rTMS were associated with reproducible within-subject changes in tinnitus severity and psychiatric symptoms over time. Findings are descriptive and hypothesis-generating and do not imply causal inference or population-level generalizability.

## Methods

This retrospective case study reports a patient to whom three identical rTMS courses were administered over a one-year period, allowing within-subject evaluation of symptom trajectories and treatment reproducibility over time. Review of the patient’s medical records indicated that no psychotropic medications or otorhinolaryngological (ENT-related) pharmacological treatments had been used for at least six months prior to the initiation of rTMS, and no pharmacological interventions were introduced during the observation period. This medication-free interval allowed for a clearer attribution of observed clinical changes to the rTMS intervention.

Clinical data were retrospectively analyzed at baseline, during, and following the rTMS courses using multidimensional outcome measures, including validated tinnitus symptom scales, psychiatric symptom ratings, and audiometric evaluations. Given the retrospective single-subject design, the analytical approach focused on within-subject longitudinal changes and clinically meaningful patterns rather than inferential between-group comparisons.

### Patient and clinical background

The patient was a 30-year-old right-handed female with an associate degree, presenting with chronic, severe, and functionally impairing tinnitus. Tinnitus onset followed a flu-like illness and was initially characterized by bilateral aural fullness and transient hearing reduction. Symptoms worsened after an ear-cleaning procedure performed at an outside center, evolving into a debilitating condition that significantly impaired quality of life. Over time, tinnitus became predominantly left-sided, while right-sided symptoms gradually diminished. Tinnitus was persistent, present both day and night, and caused marked interference with concentration and occupational functioning.

Before applying to our institution, the patient had received multiple standard and adjunctive interventions for tinnitus without sustained clinical benefit. Initial treatment included a 15-day course of systemic intravenous corticosteroids and intratympanic 5-steroid injections, neither of which provided benefit. The patient then underwent 22 sessions of hyperbaric oxygen therapy, which partially improved hearing but had no effect on tinnitus severity, followed by 10–15 sessions of a biolaser intervention, also without benefit. In an ongoing attempt to find relief, the patient subsequently tried chiropractic treatment (five sessions), two sessions of scalp hijama (wet cupping), kinesio-taping, and acupuncture with needles at other centers, none of which were helpful. A trial of sound desensitisation therapy with a hearing aid yielded no benefit. Upon presentation to our institution, a comprehensive otorhinolaryngological evaluation at baseline, including otoscopic examination and temporal magnetic resonance imaging, revealed no structural abnormalities. Despite this comprehensive work-up, no formal otological diagnosis (such as sudden sensorineural hearing loss or eustachian tube dysfunction) was established, and the aetiology of the tinnitus remained undetermined. The patient reported multiple concurrent tinnitus percepts in the left ear, including a low-pitched humming sound, a high-pitched ringing, and an electric-like noise, occasionally accompanied by an abnormal somatic sensation on the left side of the head. No associated pain or clinically significant vestibular symptoms were reported. Baseline audiometric assessment initially showed predominantly low-frequency tinnitus perception, with additional high-frequency components also reported. From an audiometric perspective, baseline evaluation demonstrated the hearing thresholds within the normal range bilaterally, with values near the upper limit of normal in the left ear, and preserved speech perception.

At baseline, the patient presented with marked depressive mood and hopelessness attributed to severe tinnitus, impaired concentration, and occupational dysfunction. Symptoms led to unpaid leave and voluntary resignation, with limited engagement in behavioral strategies and consideration of rural withdrawal if no improvement occurred.

The patient had no psychiatric, neurological, or medical history, no substance use, and no relevant family history, and remained free of psychotropic and ENT medications for six months before and throughout rTMS follow-up.

### Intervention (rTMS protocol)

No standardized rTMS protocol for tinnitus exists due to clinical heterogeneity and limited evidence; guidelines consider neuromodulation exploratory and individualized (8). Nevertheless, recent meta-analyses consistently support low-frequency (1 Hz) auditory cortex rTMS as the most studied approach, yielding modest but reproducible short-term tinnitus improvements (1, 4, 5).

Accordingly, and given the absence of an identifiable peripheral cause, a low-frequency temporoparietal rTMS protocol was selected to modulate auditory cortical hyperexcitability, which is considered a core neurophysiological mechanism underlying chronic tinnitus (9). It should be noted that auditory cortical hyperexcitability was not directly verified in this patient; it was assumed on the basis of group-level neurophysiological evidence rather than established through individual neurophysiological profiling, and the rTMS target was therefore selected on mechanistic rather than patient-specific grounds. In this case, rTMS was applied over the left auditory cortex, located at the midpoint between T3 and T5 according to the international 10–20 EEG system (10), delivered using an eight-shaped coil directed tangentially to the scalp.

**Figure 1:**
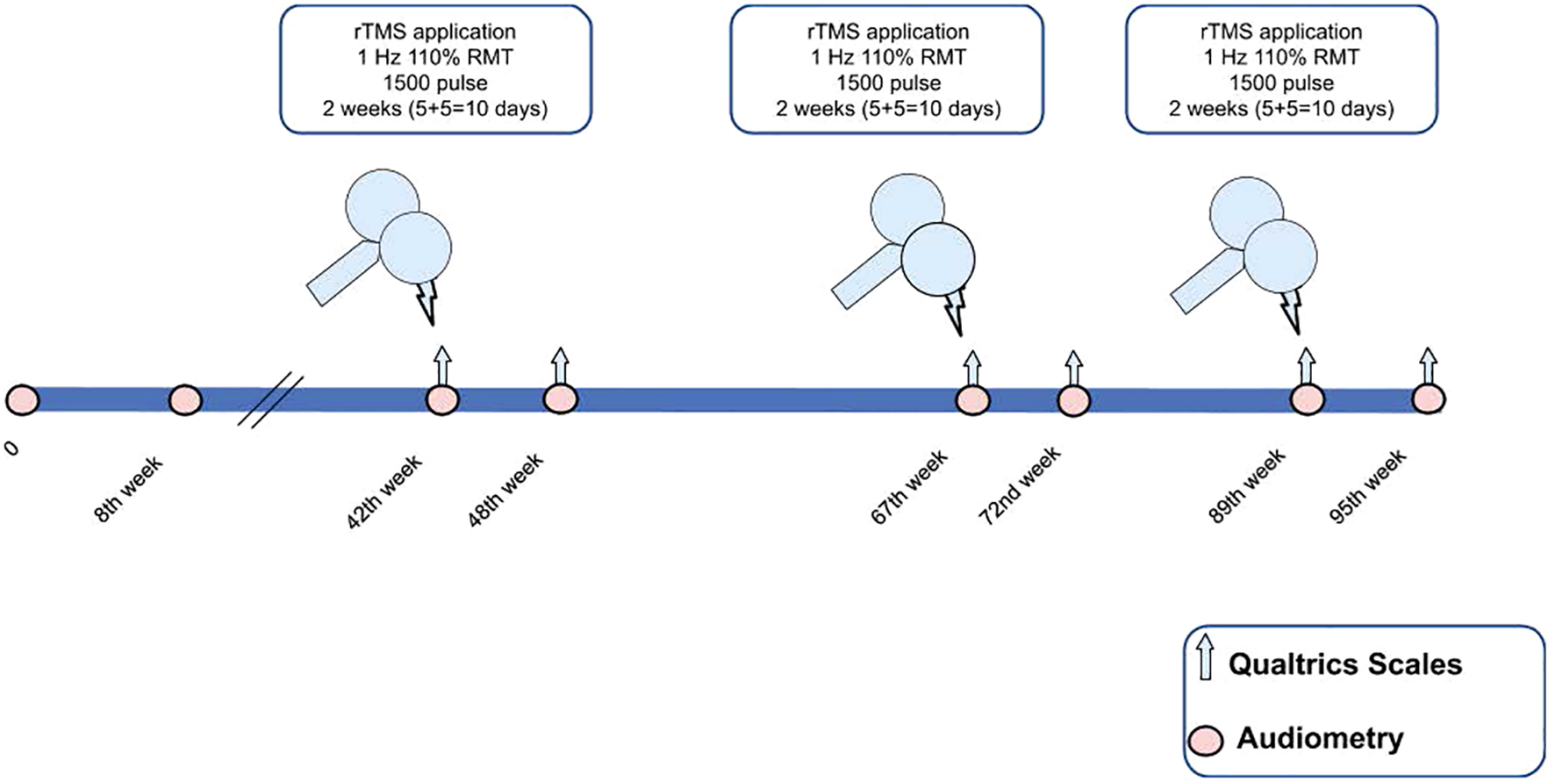
Timeline of the repetitive transcranial magnetic stimulation (rTMS) intervention and assessment schedule. rTMS was administered at 1 Hz, 110% of the resting motor threshold (RMT), with 1500 pulses per session over 2 weeks (5 + 5 = 10 days) at three separate time points. Audiometric assessments (pink circles) were conducted at baseline and follow-up visits, whereas Qualtrics-based self-report scales (upward arrows) were administered at the indicated weeks.

The protocol consisted of continuous low frequency rTMS at 1 Hz applied at 110% of the individual resting motor threshold (RMT), with a total of 1,500 pulses per session over approximately 25 minutes. Sessions were conducted five days per week for two weeks, with a total of 10 sessions administered throughout three separate occasions over a one-year period. The timing and intervals of the rTMS sessions are shown in **Figure 1**.

### Outcome measures and assessments

Assessments were conducted at predefined time points before, during, and after rTMS interventions, allowing for the evaluation of symptoms over time within subjects.

Self-report questionnaires were administered via the secure Qualtrics platform.

Consistent with the standard procedure used for online self-report scales in our clinic, attention-check items were embedded to help clinicians identify careless or inattentive responding and thereby support the validity of the self-report data. The assessment battery included a Sociodemographic Data Form, a Clinical Information Form, and validated psychometric instruments. Sociodemographic variables comprised age, gender, education, marital and employment status, and income. Clinical data covered tinnitus onset, duration, laterality, pattern, hearing loss, prior treatments, symptom severity, daily frequency, comorbid medical and psychiatric conditions, and family history of tinnitus.

Primary outcomes focused on tinnitus-related severity and functional impact. Tinnitus-related disability was assessed using the Tinnitus Handicap Inventory (THI) (11, 12), a 25-item self-report measure evaluating the functional, emotional, and catastrophic dimensions of tinnitus-related distress, with item scores of 0 (no), 2 (sometimes), and 4 (yes), yielding a total score range of 0 to 100. For clinical interpretation, total THI scores were additionally classified according to the established beta-version grading system: 0–16 (slight), 18–36 (mild), 38–56 (moderate), 58–76 (severe), and 78–100 (catastrophic). Overall tinnitus severity and its impact on daily functioning were additionally assessed using the Tinnitus Severity Index (TSI), a 12-item self-report measure of perceived tinnitus severity. As the original index was reported only in abstract form (13) but has since been used in a peer-reviewed study (14), the peer-reviewed Turkish validation (15) is cited as the validation reference. Each item is rated on a 5-point Likert scale ranging from 1 (never) to 5 (always), yielding a total score range of 12 to 60. For descriptive clinical interpretation, total TSI scores were categorized as mild (13–24), moderate (25–36), severe (37–48), and catastrophic (49–60). Subjective tinnitus intensity was measured using a single-item visual analog scale (VAS) ranging from 0 (no tinnitus) to 10 (extreme tinnitus severity) (16).

Secondary outcomes included psychological symptom burden and stress measures. Depressive symptoms were assessed using the Patient Health Questionnaire-9 (PHQ-9) (17), a nine-item self-report scale scored from 0 to 3, yielding a total score range of 0 to 27, with scores categorized as minimal (0–4), mild (5–9), moderate (10–14), moderately severe (15–19), and severe (20–27), using a validated Turkish version (18). Anxiety symptoms were measured using the Generalized Anxiety Disorder-7 (GAD-7), (19), a seven-item scale scored from 0 to 3, yielding a total score range of 0 to 21, with scores categorized as minimal (0–4), mild (5–9), moderate (10–14), and severe (15–21), using a validated Turkish version (20). Chronic stress load was assessed using the 51-item Chronic Stress Scale (21), which evaluates cumulative ongoing stressors across multiple life domains. Total scores range from 0 to 102, with higher scores indicating greater stress burden. The Turkish validity and reliability were established by Yapici-Eser et al. (22). General psychological distress was assessed using the Symptom Checklist-90-Revised (SCL-90-R) (23, 24). Perceived stress levels were additionally captured using the Stress Thermometer, a single-item VAS ranging from 0 (no stress) to 10 (extreme stress) (25).

### Audiometric assessment

An audiological evaluation was performed to determine hearing thresholds and auditory characteristics related to tinnitus over time. The hearing evaluation included pure-tone audiometry and speech audiometry, conducted in accordance with standard clinical procedures.

Pure-tone hearing thresholds were obtained using headphones with frequency-modulated stimuli delivered via a calibrated clinical audiometer (AC40 Clinical Audiometer, Interacoustic, Denmark). Thresholds were measured at frequencies ranging from 125 to 8000 Hz. The patient was instructed to indicate tone perception by pressing a response button, and thresholds were determined using a descending method in which stimulus intensity was gradually reduced until the tone was no longer perceived. Speech audiometry was performed to evaluate speech perception abilities.

In addition to audiometric thresholds, the pitch and loudness characteristics of tinnitus were assessed at each follow-up visit. The perceived frequency and intensity of low-pitched and high-pitched tinnitus components were determined based on the patient’s subjective matching and feedback during the audiometric assessment. These measurements were used descriptively to track longitudinal changes in tinnitus characteristics over time.

The case study was approved by Koç University Ethics Committee (Number:2025.444.IRB2.199), conducted in accordance with the principles of the Declaration of Helsinki, and written informed consent was obtained from the patient.

### Statistical analysis

Given the single-subject, repeated-measures design and the absence of a control condition, analyses focused on within-subject changes across repeated rTMS courses and over long-term follow-up. Outcomes were evaluated using descriptive statistics and effect size-based approaches rather than hypothesis-testing analyses.

Changes in tinnitus-related and psychological outcome measures were quantified using standardized mean change (SMC), an effect-size index based on normative standard deviations, as the primary metric. SMC was calculated as the difference between post- and pre-intervention scores divided by the normative standard deviation of each scale: SMC = (Post-Pre)/(SD_norm_)​. Normative standard deviations were derived from published validation studies and are reported in the footnotes of the corresponding tables (**Tables 1**, **2**). Normative rather than within-subject standard deviations were used, as variance estimates based on a single subject are inherently unstable and may lead to misleading effect size estimates. This approach allows effect size interpretation relative to population-level variability while avoiding inferential claims. In addition, test–retest reliability parameters required for reliable change calculations are not consistently available or applicable across several of the original validation studies of the instruments used. Negative SMC values indicate symptom improvement, whereas positive values indicate symptom worsening. Effect size magnitude was interpreted according to conventional thresholds: trivial (<0.20), small (≥0.20), moderate (≥0.50), and large (≥0.80) (26).

**Table 1:** Episode-based and overall within-subject changes across tinnitus outcome measures.

Scale	Comparison	Week (Pre)	Pre	Week (Post)	Post	Δ (Post–Pre)	% Change	SMC (d)	Effect size
Tinnitus Handicap Inventory	Pre-post rTMS 1	42	98	48	90	−8	−8.2%	−0.39	Small
	Pre-post rTMS 2	67	56	72	66	+10	+17.9%	+0.49	Small
	Pre-post rTMS 3	89	70	95	50	−20	−28.6%	−0.98	Large
	Overall (Baseline→Final)	42	98	95	50	−48	−49.0%	−2.34	Large
Tinnitus Severity Index	Pre-post rTMS 1	42	59	48	57	−2	−3.4%	−0.22	Small
	Pre-post rTMS 2	67	44	72	46	+2	+4.5%	+0.22	Small
	Pre-post rTMS 3	89	40	95	41	+1	+2.5%	+0.11	Trivial
	Overall (Baseline→Final)	42	59	95	41	−18	−30.5%	−2.00	Large
VAS-Tinnitus Intensity	Pre-post rTMS 1	42	10	48	8	−2	−20.0%	−0.80	Large
	Pre-post rTMS 2	67	7	72	6	−1	−14.3%	−0.40	Small
	Pre-post rTMS 3	89	6	95	5	−1	−16.7%	−0.40	Small
	Overall (Baseline→Final)	42	10	95	5	−5	−50.0%	−2.00	Large

Standardized mean change (SMC) was calculated as (*Post-Pre*)/(*SD_norm_*). Normative standard deviations (*SD_norm_*) used in the calculations were 20.5 for the Tinnitus Handicap Inventory (12), 9.00 for the Tinnitus Severity Index (14), and 2.5 for the visual analogue scale (VAS) for tinnitus (16). Negative SMC values indicate improvement, whereas positive values indicate worsening. Effect size magnitude was interpreted according to conventional thresholds: trivial (<0.20), small (≥0.20), moderate (≥0.50), and large (≥0.80) (26).

**Table 2:** Episode-based and overall within-subject changes across psychological outcome measures.

Scale	Comparison	Week (Pre)	Pre	Week (Post)	Post	Δ (Post–Pre)	% Change	SMC (d)	Effect size
PHQ-9 (Depression)	Pre-post rTMS 1	42	15	48	6	−9	−60.0%	−1.72	Large
	Pre-post rTMS 2	67	3	72	3	0	0.0%	0.00	Trivial
	Pre-post rTMS 3	89	5	95	2	−3	−60.0%	−0.57	Moderate
	Overall (Baseline→Final)	42	15	95	2	−13	−86.7%	−2.49	Large
GAD-7 (Anxiety)	Pre-post rTMS 1	42	13	48	1	−12	−92.3%	−2.76	Large
	Pre-post rTMS 2	67	0	72	0	0	0.0%	0.00	Trivial
	Pre-post rTMS 3	89	0	95	0	0	0.0%	0.00	Trivial
	Overall (Baseline→Final)	42	13	95	0	−13	−100%	−2.99	Large
CSS (Stress Load)	Pre-post rTMS 1	42	17	48	6	−11	−64.7%	−0.88	Large
	Pre-post rTMS 2	67	5	72	6	+1	+20.0%	+0.08	Trivial
	Pre-post rTMS 3	89	6	95	6	0	0.0%	0.00	Trivial
	Overall (Baseline→Final)	42	17	95	6	−11	−64.7%	−0.88	Large
VAS-Stress (0–10)	Pre-post rTMS 1	42	10	48	7	−3	−30.0%	−1.24	Large
	Pre-post rTMS 2	67	4	72	4	0	0.0%	0.00	Trivial
	Pre-post rTMS 3	89	5	95	3	−2	−40.0%	−0.83	Large
	Overall (Baseline→Final)	42	10	95	3	−7	−70.0%	−2.90	Large

Standardized mean change (SMC) was calculated as (*Post-Pre*)/(*SD_norm_*) Normative standard deviations (*SD_norm_*) used in the calculations were 5.23 for the Patient Health Questionnaire-9 (PHQ-9) (18), 4.35 for the Generalized Anxiety Disorder-7 (GAD-7) (20), 12.54 for the Chronic Stress Scale (CSS) (22), and 2.41 for the visual analogue scale for stress (VAS-Stress; 0–10) (25). Negative SMC values indicate improvement, whereas positive values indicate worsening. Effect size magnitude was interpreted according to conventional thresholds: trivial (<0.20), small (≥0.20), moderate (≥0.50), and large (≥0.80) (26).

Analyses were conducted at two levels. First, episode-based analyses compared pre- and post-treatment scores for each rTMS course to characterize short-term effects. Second, overall analyses compared baseline and final follow-up scores to evaluate cumulative long-term effects across the entire treatment period. Percentage change from pre- to post-intervention was additionally calculated to facilitate clinical interpretation.

Due to heterogeneity in reliability reporting across the included measures and to maintain a conservative analytic strategy, reliable change indices and formal statistical significance testing were not performed. Accordingly, conclusions were based on the magnitude, direction, and internal consistency of standardized mean changes, thereby emphasizing robust patterns of within-subject change while minimizing the risk of overinterpretation in a single-subject design.

SCL-90-R outcomes were displayed exclusively in graphical form to illustrate longitudinal changes in overall psychological distress. Audiometric data were analyzed descriptively. Changes in tinnitus pitch and perceived loudness were summarized longitudinally to characterize qualitative and quantitative shifts in tinnitus perception over time.

## Results

### Tinnitus severity outcomes

**Figure 2:**
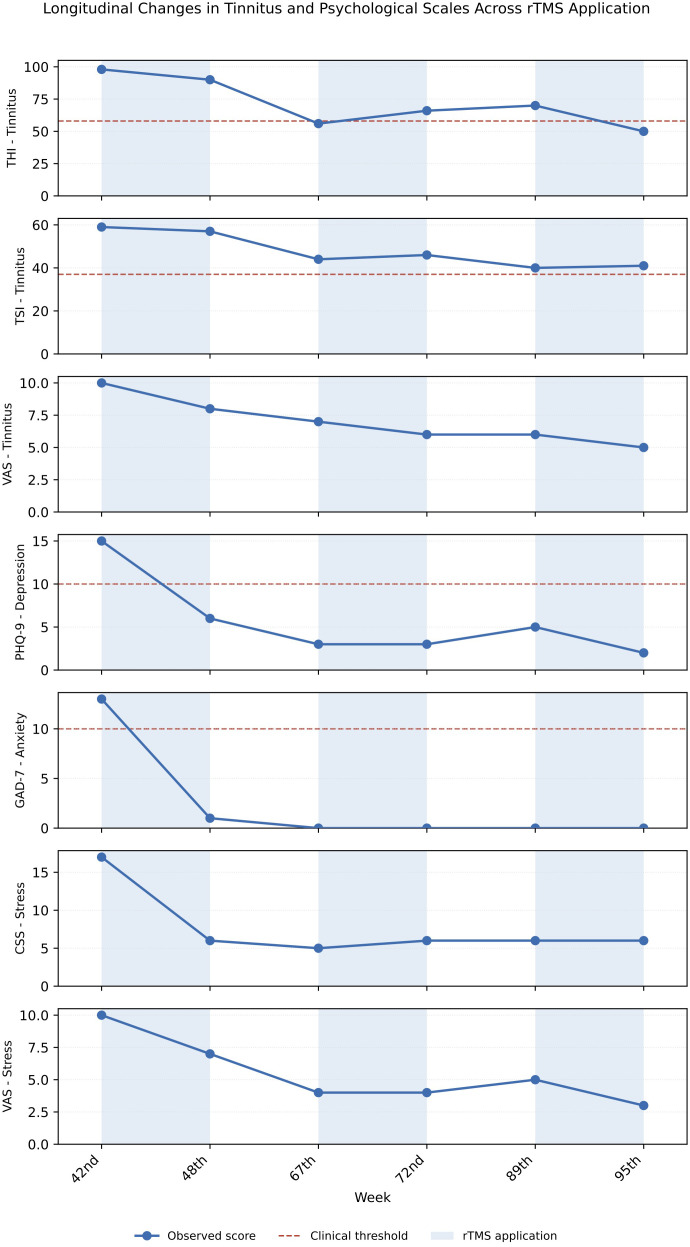
Longitudinal changes in tinnitus-related and psychological outcome measures across the rTMS treatment course. Line plots illustrate observed scores across assessment weeks (42nd–95th), each measure shown on its own zero-based axis. Shaded vertical bands indicate periods of rTMS application. Horizontal dashed lines denote clinically meaningful thresholds: the moderate/severe boundary for the THI (58) and TSI (37), and the moderate-symptom threshold for the PHQ-9 (10) and GAD-7 (10). No validated clinical cut-off exists for the single-item VAS measures or the Chronic Stress Scale, and reference lines were therefore not drawn for these. THI, Tinnitus Handicap Inventory; TSI, Tinnitus Severity Index; VAS, Visual Analog Scale; PHQ-9, Patient Health Questionnaire-9; GAD-7, Generalized Anxiety Disorder-7; CSS, Chronic Stress Scale; rTMS, repetitive Transcranial Magnetic Stimulation.

**Table 1** summarizes episode-based and overall within-subject changes across tinnitus-related outcome measures. Longitudinal changes in tinnitus-related outcome measures across the three rTMS courses and follow-up period are additionally illustrated in **Figure 2**.

Across each rTMS course, THI demonstrated heterogeneous short-term effects, with a small improvement following the first course (SMC = −0.39), a small worsening after the second course (SMC = +0.49), and a large improvement after the third course (SMC = −0.98). Overall, THI decreased from 98 at baseline to 50 at the final assessment, corresponding to a large overall effect (SMC = −2.34).

For TSI, short-term effects across individual rTMS courses were small to trivial (SMC range: −0.22 to +0.22). In contrast, the overall comparison showed a reduction from 59 at baseline to 41 at the final assessment, yielding a large overall effect (SMC = −2.00).

VAS tinnitus intensity showed consistent short-term improvements across all rTMS courses, with effect sizes ranging from small to large (SMC = −0.40 to −0.80). Overall, VAS scores declined from 10 at baseline to 5 at the final assessment, corresponding to a large overall effect (SMC = −2.00).

### Psychological symptom outcomes

**Table 2** summarizes episode-based and overall within-subject changes across psychological outcome measures following three repeated rTMS courses.

Across psychological outcome measures, symptom levels decreased substantially following the first rTMS course, reaching low or near floor-level values, such that no further short-term changes were observed during subsequent courses. Consistent with this pattern, depressive symptoms (PHQ-9) showed a large short-term improvement after the first course (SMC = −1.72), no change following the second course (SMC = 0.00), and a moderate improvement after the third course (SMC = −0.57). Overall, PHQ-9 scores decreased from 15 at baseline to 2 at the final assessment, corresponding to a large overall effect (SMC = −2.49), driven predominantly by the initial rTMS course, with scores reaching floor level thereafter.

Similarly, anxiety symptoms (GAD-7) demonstrated a large short-term improvement after the first rTMS course (SMC = −2.76), whereas no short-term changes were observed following the second and third courses (SMC = 0.00 for both), consistent with floor-level baseline scores. The overall comparison indicated a complete reduction in anxiety symptoms from baseline to final assessment (13 to 0; SMC = −2.99); as GAD-7 scores were already at floor level before the second and third courses, this reflects maintenance of the initial-course improvement rather than an additional contribution of the later courses.

For stress load (CSS), a large reduction was observed following the first rTMS course (SMC = −0.88), with trivial changes during the second and third courses (SMC = +0.08 and 0.00, respectively). Despite this variability, the overall comparison demonstrated a sustained reduction from baseline to final assessment (17 to 6; SMC = −0.88), again driven by the initial course and maintained thereafter rather than accruing across courses.

Finally, VAS-rated stress intensity showed a large short-term reduction after the first rTMS course (SMC = −1.24), no change following the second course (SMC = 0.00), and a large reduction after the third course (SMC = −0.83). Overall, VAS-Stress scores declined from 10 at baseline to 3 at the final assessment, (SMC = −2.90), predominantly reflecting the initial-course reduction, with a further reduction during the third course.

**Figure 3:**
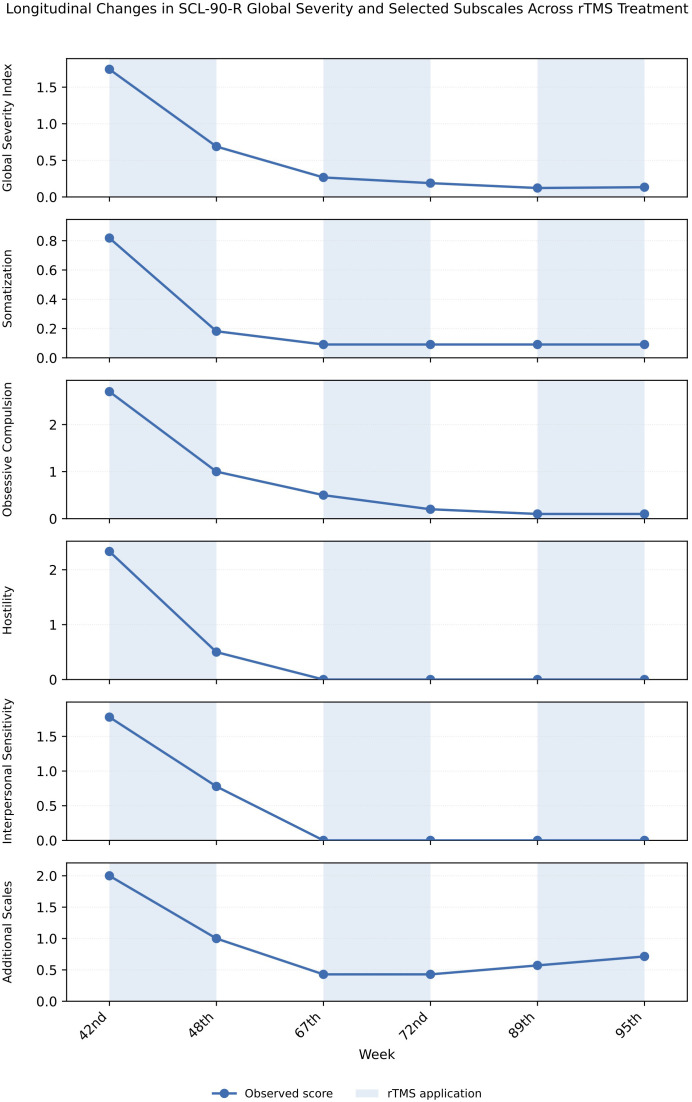
Longitudinal changes in the symptom checklist-90-revised global severity index and selected subscale scores across the rTMS treatment course. Line plots illustrate observed scores at each assessment week. Shaded vertical bands indicate periods of rTMS administration. SCL-90-R, Symptom Checklist-90-Revised; rTMS, repetitive transcranial magnetic stimulation.

Changes in global psychological distress were examined in greater detail using the Symptom Checklist-90-Revised (SCL-90-R). Longitudinal assessment revealed a meaningful and sustained decrease in overall symptom burden throughout the follow-up period. Decreases were observed in the Global Severity Index and in symptom dimensions (**Figure 3**).

### Audiometric outcomes

The baseline audiometric assessment performed prior to rTMS initiation showed that hearing thresholds were within the normal range in both ears. Speech audiometry demonstrated preserved speech perception with high speech discrimination performance in both ears. In all post-rTMS assessments, pure tone thresholds and speech audiometric measurements remained within a narrow range without any sign of progressive deterioration, demonstrating stable peripheral hearing sensitivity throughout the observation period.

Initially, the low-pitched tinnitus “humming” was clearly more noticeable during the early follow-up visits and may have masked the high-pitched “ringing” component. As the low-pitched tinnitus gradually improved with TMS, the patient started to become aware of the high-pitched sound as well. In the later visits, both components showed a reduction in intensity. Pure-tone and speech audiometry measures remained stable across all follow-up assessments. This stability effectively serves as a negative control, suggesting that observed improvements in tinnitus-related outcomes were not accompanied by measurable changes in peripheral hearing function. Tinnitus pitch and loudness matching was performed descriptively based on the patient’s subjective report and audiometric feedback, and the results were analyzed qualitatively rather than as precise quantitative measures.

## Discussion

In the present case, changes in tinnitus perception were observed across longitudinal follow-up, with reductions in the perceived intensity of tinnitus components over time.

Consistent with these qualitative changes, the longitudinal data demonstrated a coherent pattern of reduction in the patient’s subjectively perceived tinnitus characteristics, including perceived sound quality, intensity, and overall tinnitus-related distress. Over the same observation period, depression and anxiety symptoms also showed a notable reduction in the absence of concurrent changes in psychiatric treatment beyond routine clinical evaluations. Symptom improvement was most pronounced during the period of the initial rTMS course, whereas subsequent courses were primarily characterized by the persistence of previously observed symptom reductions, with little additional short-term improvement.

The magnitude of observed symptom change during the initial rTMS course may be partly attributable to the high baseline severity of tinnitus, which may allow for a broader dynamic range for observable change (27). Following the initial course, subsequent courses were characterized by neither rebound nor further short-term improvement. Because no control condition was included, this pattern cannot be interpreted as treatment-induced stabilization; it is equally consistent with a floor effect after a large initial change, and causal attribution to the repeated courses is not possible. This floor-related pattern applies mainly to the psychiatric measures (PHQ-9, GAD-7, and the stress measures), which reached low or floor-level values after the initial course; their large overall effect sizes therefore reflect maintenance of the initial-course improvement rather than a cumulative contribution of the later courses. While prior studies, including long-term follow-ups, have shown that the clinical benefits of rTMS for tinnitus may attenuate over time following a single treatment course, relatively little information is available regarding the clinical course of patients receiving repeated courses of the same rTMS protocol over time (1, 5). In this context, the sustained symptom reductions observed across approximately one year of follow-up in the present single-subject case may plausibly coincide with repeated courses of rTMS applications over time, although causal inference cannot be established. In contrast, the tinnitus-specific outcomes did not reach floor level and retained an interpretive window across all three courses. The THI improved slightly after the first course, worsened transiently after the second alongside increased stress and depressive symptoms, and improved markedly after the third, while VAS-Tinnitus improved consistently. As these changes are not floor-driven, the between-course dynamics of the THI and VAS are more informative than the psychiatric trajectories regarding the contribution of repeated administration, consistent with state-dependent modulation of tinnitus perception rather than a monotonic response.

An important observation from the longitudinal data is that during the second rTMS course, short-term changes in THI and TSI scores were small or trivial in magnitude, coinciding with a transient increase in stress and depressive symptoms related to concurrent life stressors. This pattern is consistent with the possibility that tinnitus severity may be dynamically influenced by affective and stress-related states, even during ongoing neuromodulatory intervention. This interpretation is consistent with network-based models conceptualizing tinnitus as a disorder involving interactions between auditory cortical hyperexcitability and limbic-stress systems (9). A prior study suggests that affective symptom burden, including depressive features, may transiently influence perceived clinical response to rTMS without necessarily reflecting a loss of neuromodulatory efficacy (28). Importantly, alongside improvements in depression, anxiety, and stress measures, a sustained reduction was observed in the SCL-90-R Global Severity Index and across multiple symptom dimensions, indicating a generalized attenuation of psychological distress that may have contributed, at least in part, to changes in tinnitus perception. Accordingly, fluctuations in stress and depressive symptoms may be clinically relevant when interpreting longitudinal tinnitus outcomes in neuromodulation studies, given the established influence of affective comorbidities on tinnitus distress (8). These findings underscore the potential dissociation between auditory measures and subjective clinical outcomes, while emphasizing the role of repeated, well-monitored neuromodulation interventions in understanding symptom trajectories and psychiatric changes over the observation period. An additional finding was the stability of audiometric thresholds and speech perception measures throughout the observation period. Despite clinically meaningful reductions in tinnitus-related distress and perceived tinnitus intensity, no measurable changes were observed in peripheral hearing function. This pattern is consistent with contemporary models proposing that rTMS primarily modulates central auditory, attentional, and limbic networks rather than peripheral auditory mechanisms. The dissociation between stable audiometric measures and reduced tinnitus severity further supports the view that subjective tinnitus burden may improve independently of changes in hearing thresholds. Notably, as the initially dominant low-pitched tinnitus component became less prominent over time, the patient reported increased awareness of a previously less noticeable high-pitched component before both percepts subsequently declined in intensity. Although this observation may reflect changes in the relative perceptual salience of different tinnitus components, these pitch- and loudness-matching findings were based on subjective reports and should therefore be interpreted cautiously.

Across the repeated rTMS courses administered over approximately one year, no adverse effects were observed. Sessions conducted at long intervals were well tolerated by the patient, and no cumulative or delayed adverse events were reported during follow-up. These observations are consistent with the feasibility and tolerability of repeated rTMS courses and underscore the need for systematic evaluation in controlled longitudinal studies of treatment-resistant tinnitus.

A previous randomized, sham-controlled study employing a comparable 4-week low-frequency rTMS protocol targeting the temporoparietal junction reported no significant benefit compared with sham stimulation. However, the study was conducted in a small parallel-group sample and included patients with long-standing chronic tinnitus, which may have limited the observable treatment response (29). Prolonged tinnitus duration is likely associated with more entrenched maladaptive neuroplastic changes, potentially reducing responsiveness to neuromodulator interventions. In contrast, greater baseline tinnitus severity, as reflected by higher THI scores, may provide a wider therapeutic window, allowing for more pronounced measurable improvement. This finding has also been seen in another study where higher tinnitus severity was associated with altered neurobiological responses to rTMS (30). Taken together, these findings suggest that both disease chronicity and baseline symptom severity may critically influence the effect size of rTMS, highlighting the importance of careful patient stratification when interpreting treatment outcomes. rTMS may be associated with changes in tinnitus perception within large-scale functional networks, as suggested by studies demonstrating associations between changes in functional connectivity and reductions in tinnitus severity (31). In this context, incomplete remission may reflect a reduced perceptual prominence of the tinnitus signal, such as diminished attentional capture or emotional reactivity, rather than complete elimination of the underlying tinnitus-generating neural activity (32).

Beyond neurobiological mechanisms, the clinical expression and course of tinnitus are also shaped by higher-order cognitive and psychiatric processes, particularly attentional focus and the meaning attributed to the tinnitus percept (33). Patients with chronic tinnitus often demonstrate increased attentional allocation to the tinnitus percept, which may amplify the perceived severity of symptoms (34). Importantly, such changes in perception may occur even in the absence of complete elimination of the underlying auditory signal (33). This view is consistent with established neurophysiological and cognitive models of tinnitus, which emphasize the role of attention, perceptual salience, and emotional appraisal in shaping the subjective experience of tinnitus (34, 35).

As limitations, given the single-subject design, the large, standardized effect sizes observed in this case should be interpreted cautiously and should not be viewed as indicators of population-level treatment efficacy. Although this patient benefited from close longitudinal monitoring and the use of multiple validated psychometric and functional scales, its interpretability is limited by the single-patient design, which inherently restricts generalizability and precludes causal conclusions. A further limitation is that the observed improvements cannot be separated from expectancy-related and other non-specific treatment effects. The patient had previously undergone numerous unsuccessful interventions and was subsequently enrolled in a novel, closely monitored research protocol with frequent clinical contact, a context that may itself foster therapeutic expectation. The catastrophic baseline THI score additionally allowed substantial room for regression toward the mean and for expectation-driven change. In the absence of a sham-controlled condition, the contribution of these non-specific factors to the observed outcomes cannot be determined, and the findings should be interpreted accordingly. In addition, while substantial symptom reduction was observed following the initial rTMS course, subsequent treatment cycles were primarily characterized by symptom stabilization rather than further short-term improvement, suggesting potential floor effects and state-dependent variability in treatment response. The absence of advanced objective neurobiological measures, such as functional magnetic resonance imaging or other neurophysiological assessments, limits the ability to directly link observed clinical improvements to underlying central auditory or cortical network changes and constrains mechanistic interpretation. While the findings are not intended to support population-level generalization, the longitudinal N-of-1 design offers a unique opportunity to examine within-subject reproducibility and contextual modulation of rTMS effects. Such designs may contribute to the conceptual development of adaptive, repeated-course neuromodulation strategies relevant to individualized treatment approaches.

Another limitation concerns the repeated administration of these instruments over one year, which has not been formally validated. The self-report scales were established primarily in cross-sectional studies and may be subject to practice, recall, and habituation effects; for most of them, repeated-administration stability has not been investigated, and even for the most studied measures, longitudinal measurement invariance has not been demonstrated for the PHQ-9 and GAD-7 (36), implying that score differences may not reflect change on a fully equivalent metric. We therefore interpreted these scores as within-subject trajectories rather than psychometrically calibrated change values. In contrast, the audiometric assessments are objective, standardized procedures; pure-tone audiometry is the gold standard for hearing sensitivity, with test-retest repeatability such that a shift exceeding approximately 10 dB is required to indicate a true change (37). Here, pure-tone and speech thresholds remained well within this range across all visits, serving as an internal negative control rather than a primary outcome.

## Conclusion

This retrospective N-of-1 study suggests that repeated courses of low-frequency rTMS may be associated with sustained reductions in tinnitus severity and with improvements in psychiatric symptoms over a one-year observation period. Variations in tinnitus-related outcomes appeared to coincide with fluctuations in affective and stress-related states, consistent with contemporary network-based models emphasizing interactions between auditory and limbic-stress systems. Given the single-subject design and absence of a control condition, these observations should be considered hypothesis-generating. Further controlled studies are needed to evaluate the potential role of repeated courses of rTMS in chronic tinnitus.

## Data Availability

The raw data supporting the conclusions of this article will be made available by the authors, without undue reservation.
